# (*E*)-Ethyl 3-(4-fluoro­anilino)-2-(4-methoxy­phen­yl)acrylate

**DOI:** 10.1107/S1600536808039184

**Published:** 2008-11-26

**Authors:** Da-Gui Zheng, Zhu-Ping Xiao

**Affiliations:** a Key Laboratory of Applied Organic Chemistry, Higher Institutions of Jiangxi Province, Shangrao Normal College, Shangrao 334001, Jiangxi, People’s Republic of China; b College of Chemistry & Chemical Engineering, Jishou University, Jishou 416000, People’s Republic of China

## Abstract

In the title compound, C_18_H_18_FNO_3_, the dihedral angles between the two benzene rings and the plane through the acrylate group and the fluoro­phenyl ring are 61.58 (8) and 13.33 (9)°, respectively. Mol­ecules are linked into ribbons through C—H⋯O and N—H⋯O hydrogen bonds, and further linked by C—H⋯π inter­actions, forming a three-dimensional network.

## Related literature

For related literature regarding the anti­microbial activity of 3-aryl­amino-2-aryl acrylates, see: Shi *et al.* (2007 ▶); Xiao *et al.* (2007 ▶, 2008 ▶); Xue *et al.* (2007 ▶). For bond-length data, see: Allen *et al.* (1987 ▶).
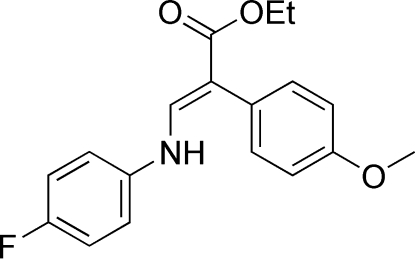


## Experimental

### 

#### Crystal data

C_18_H_18_FNO_3_
*M* *_r_* = 315.33
Monoclinic, 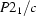

*a* = 19.000 (4) Å
*b* = 6.0400 (12) Å
*c* = 15.081 (3) Å
β = 109.64 (3)°
*V* = 1630.0 (6) Å^3^
*Z* = 4
Mo *K*α radiation
μ = 0.10 mm^−1^
*T* = 293 (2) K
0.30 × 0.30 × 0.20 mm

#### Data collection

Enraf–Nonius CAD-4 diffractometer
Absorption correction: ψ scan (North *et al.*, 1968 ▶) *T*_min_ = 0.972, *T*_max_ = 0.981
3073 measured reflections
2943 independent reflections
1807 reflections with *I* > 2σ(*I*)
*R*_int_ = 0.026

#### Refinement

*R*[*F* ^2^ > 2σ(*F* ^2^)] = 0.050
*wR*(*F* ^2^) = 0.162
*S* = 1.02
2943 reflections
215 parameters
H atoms treated by a mixture of independent and constrained refinement
Δρ_max_ = 0.15 e Å^−3^
Δρ_min_ = −0.16 e Å^−3^

### 

Data collection: *CAD-4 Software* (Enraf–Nonius, 1989 ▶); cell refinement: *CAD-4 Software*; data reduction: *XCAD4* (Harms & Wocadlo, 1995 ▶); program(s) used to solve structure: *SHELXS97* (Sheldrick, 2008 ▶); program(s) used to refine structure: *SHELXL97* (Sheldrick, 2008 ▶); molecular graphics: *XP* in *SHELXTL* (Sheldrick, 2008 ▶); software used to prepare material for publication: *SHELXL97*.

## Supplementary Material

Crystal structure: contains datablocks global, I. DOI: 10.1107/S1600536808039184/ez2149sup1.cif

Structure factors: contains datablocks I. DOI: 10.1107/S1600536808039184/ez2149Isup2.hkl

Additional supplementary materials:  crystallographic information; 3D view; checkCIF report

## Figures and Tables

**Table 1:** Hydrogen-bond geometry (Å, °)

*D*—H⋯*A*	*D*—H	H⋯*A*	*D*⋯*A*	*D*—H⋯*A*
C12—H12⋯O1^i^	0.93	2.49	3.401 (3)	167
N1—H1⋯O3^ii^	0.83 (2)	2.56 (3)	3.229 (3)	138 (2)
C16—H16*B*⋯*Cg*1^iii^	0.97	2.99	3.788 (3)	141
C18—H18*A*⋯*Cg*2^iv^	0.96	2.80	3.626 (3)	145

Symmetry codes: (i) 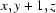; (ii) 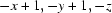; (iii) 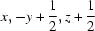; (iv) 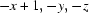. *Cg*1 and *Cg*2 are the centroids of the C1–C6 and C7–C12 rings, respectively.

**Fig. 1.**
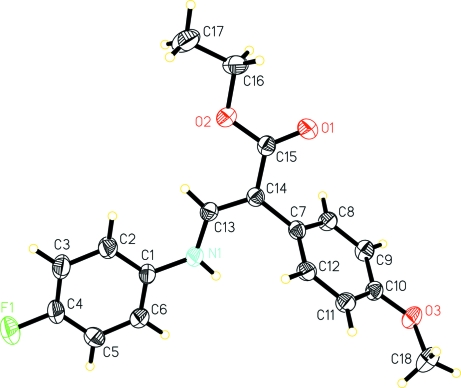
The molecular structure of the title compound, showing the atom-numbering scheme. Displacement ellipsoids are drawn at the 30% probability level.

**Fig. 2.**
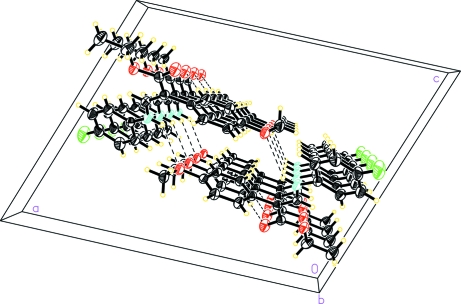
A partial packing diagram of the title compound showing the ribbons connected by C—H···O and N—H···O hydrogen bonds (indicated by dashed lines), viewed along the b axis.

**Fig. 3.**
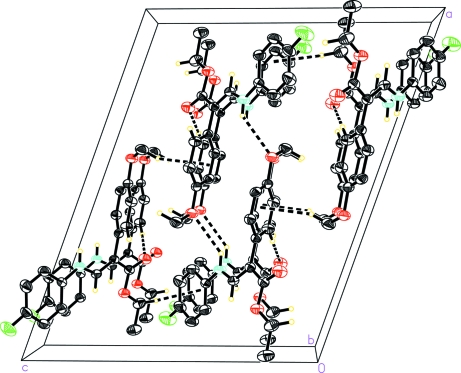
The crystal packing of the title compound, showing the linking of the hydrogen bonded ribbons by C—H···π interactions. Dashed lines indicate C—H···O, N—H···O and C-H···π interactions.

## supplementary crystallographic information

### Comment

3-Arylamino-2-aryl acrylates, enamines structurally like Schiff bases, show high antimicrobial activity (Xiao *et al.*, 2007; Xue *et al.*, 2007; Xiao *et al.*, 2008; Shi *et al.* 2007), especially for bacteria. In a continuation of our work on the structural characterization of enamine derivatives, we report here the crystal structure of the title compound, (I) (Fig. 1).

The N1—H group lies approximately in the same planes as the fluorophenyl and acrylate groups (with dihedral angles of 4.7 (2) ° and 8.9 (2) °, respectively), suggesting that one of the p orbitals of N1 is conjugated with the π molecular orbitals of the two moieties, thus shortening both the C1—N1 (1.408 (3) Å) and C13—N1 (1.359 (3) Å) bonds. All other double and single bond lengths fall within normal values (Allen *et al.*, 1987).

Molecules are linked into ribbons running along the b-axis via C—H···O and N—H···O hydrogen bonds (Fig. 2 and Table 1). These ribbons are interconnected via weak C16-H16B···π (centroid of C1 to C6) and C18-H18A···π (centroid of C7 to C12) interactions (Table 1 and Fig. 3).

### Experimental

Equimolar quantities (6 mmol) of ethyl 2-(4-methoxyphenyl)-3- oxopropanoate (1.33 g) and 4-fluorobenzenamine (0.67 g) in absolute alcohol (18 ml) were heated at 344–354 K for 2 h. The excess solvent was removed under reduced pressure. The residue was purified by flash chromatography with EtOAc-petrolum ether (1:10) to afford two fractions. The first fraction gave the *Z*-isomer, and the second fraction, after partial solvent evaporation, furnished colorless blocks of (I) suitable for single-crystal structure determination.

### Refinement

The H atom bonded to N1 was located in a difference Fourier map. All other H atoms were placed in geometrically idealized positions and constrained to ride on their parent atoms with C—H = 0.93, 0.96 and 0.97 Å for the aromatic, CH_3_ and CH_2_ type H atoms, respectively. *U*_iso_ = 1.2*U*_eq_(parent atoms) were assigned for amino, aromatic and CH_2_ type H-atoms and 1.5*U*_eq_(parent atoms) for CH_3_ type H-atoms.

### Crystal data

**Table:**

C_18_H_18_FNO_3_	*F*_000_ = 664
*M**_r_* = 315.33	*D*_x_ = 1.285 Mg m^−^^3^
Monoclinic, *P*2_1_/*c*	Mo *K*α radiation λ = 0.71073 Å
*a* = 19.000 (4) Å	Cell parameters from 1632 reflections
*b* = 6.0400 (12) Å	θ = 1.4–24.7º
*c* = 15.081 (3) Å	µ = 0.10 mm^−^^1^
β = 109.64 (3)º	*T* = 293 (2) K
*V* = 1630.0 (6) Å^3^	Block, colorless
*Z* = 4	0.30 × 0.30 × 0.20 mm

### Data collection

**Table:**

Enraf–Nonius CAD-4 diffractometer	2943 independent reflections
Radiation source: fine-focus sealed tube	1807 reflections with *I* > 2σ(*I*)
Monochromator: graphite	*R*_int_ = 0.026
*T* = 293(2) K	θ_max_ = 25.3º
ω/2θ scans	θ_min_ = 1.1º
Absorption correction: ψ scan(North *et al.*, 1968)	*h* = −22→21
*T*_min_ = 0.972, *T*_max_ = 0.981	*k* = −7→0
3073 measured reflections	*l* = 0→18

### Refinement

**Table:**

Refinement on *F*^2^	Hydrogen site location: inferred from neighbouring sites
Least-squares matrix: full	H atoms treated by a mixture of independent and constrained refinement
*R*[*F*^2^ > 2σ(*F*^2^)] = 0.050	*w* = 1/[σ^2^(*F*_o_^2^) + (0.0863*P*)^2^ + 0.0162*P*] where *P* = (*F*_o_^2^ + 2*F*_c_^2^)/3
*wR*(*F*^2^) = 0.162	(Δ/σ)_max_ < 0.001
*S* = 1.02	Δρ_max_ = 0.15 e Å^−^^3^
2943 reflections	Δρ_min_ = −0.16 e Å^−^^3^
215 parameters	Extinction correction: SHELXL97 (Sheldrick, 2008), Fc^*^=kFc[1+0.001xFc^2^λ^3^/sin(2θ)]^-1/4^
Primary atom site location: structure-invariant direct methods	Extinction coefficient: 0.044 (4)
Secondary atom site location: difference Fourier map	

### Special details

**Table:**

Geometry. All e.s.d.'s (except the e.s.d. in the dihedral angle between two l.s. planes) are estimated using the full covariance matrix. The cell e.s.d.'s are taken into account individually in the estimation of e.s.d.'s in distances, angles and torsion angles; correlations between e.s.d.'s in cell parameters are only used when they are defined by crystal symmetry. An approximate (isotropic) treatment of cell e.s.d.'s is used for estimating e.s.d.'s involving l.s. planes.
Refinement. Refinement of *F*^2^ against ALL reflections. The weighted *R*-factor *wR* and goodness of fit *S* are based on *F*^2^, conventional *R*-factors *R* are based on *F*, with *F* set to zero for negative *F*^2^. The threshold expression of *F*^2^ > σ(*F*^2^) is used only for calculating *R*-factors(gt) *etc*. and is not relevant to the choice of reflections for refinement. *R*-factors based on *F*^2^ are statistically about twice as large as those based on *F*, and *R*- factors based on ALL data will be even larger.

### Fractional atomic coordinates and isotropic or equivalent isotropic displacement parameters (Å^2^)

**Table:**

	*x*	*y*	*z*	*U*_iso_*/*U*_eq_	
C1	0.78366 (12)	0.7249 (4)	0.02705 (17)	0.0468 (6)	
C2	0.86057 (14)	0.7171 (5)	0.0575 (2)	0.0637 (8)	
H2	0.8858	0.6095	0.1005	0.076*	
C3	0.90034 (15)	0.8673 (6)	0.0246 (2)	0.0733 (9)	
H3	0.9523	0.8617	0.0451	0.088*	
C4	0.86292 (16)	1.0237 (5)	−0.0380 (2)	0.0682 (9)	
C5	0.78687 (17)	1.0339 (5)	−0.0708 (2)	0.0721 (9)	
H5	0.7620	1.1403	−0.1148	0.086*	
C6	0.74779 (15)	0.8829 (5)	−0.0373 (2)	0.0640 (8)	
H6	0.6958	0.8882	−0.0588	0.077*	
C7	0.64691 (12)	0.3267 (4)	0.14607 (16)	0.0431 (6)	
C8	0.59236 (13)	0.1688 (4)	0.10497 (18)	0.0494 (7)	
H8	0.6063	0.0338	0.0862	0.059*	
C9	0.51803 (13)	0.2090 (5)	0.09161 (18)	0.0513 (7)	
H9	0.4825	0.1013	0.0639	0.062*	
C10	0.49607 (13)	0.4075 (4)	0.11902 (17)	0.0470 (6)	
C11	0.54911 (13)	0.5661 (4)	0.16114 (19)	0.0523 (7)	
H11	0.5350	0.7002	0.1804	0.063*	
C12	0.62356 (13)	0.5233 (4)	0.17434 (19)	0.0510 (7)	
H12	0.6591	0.6302	0.2032	0.061*	
C13	0.76733 (13)	0.4059 (4)	0.12087 (17)	0.0465 (6)	
H13	0.8176	0.3699	0.1356	0.056*	
C14	0.72695 (12)	0.2824 (4)	0.16064 (16)	0.0433 (6)	
C15	0.76226 (13)	0.0959 (4)	0.22121 (18)	0.0458 (6)	
C16	0.87264 (15)	−0.1081 (5)	0.2948 (2)	0.0669 (8)	
H16A	0.8550	−0.2514	0.2671	0.080*	
H16B	0.8630	−0.0960	0.3538	0.080*	
C17	0.95460 (15)	−0.0844 (6)	0.3113 (3)	0.0887 (11)	
H17A	0.9637	−0.1045	0.2530	0.133*	
H17B	0.9817	−0.1941	0.3558	0.133*	
H17C	0.9709	0.0606	0.3358	0.133*	
C18	0.39590 (15)	0.6241 (5)	0.1368 (2)	0.0751 (9)	
H18A	0.4220	0.6374	0.2032	0.113*	
H18B	0.3432	0.6130	0.1255	0.113*	
H18C	0.4057	0.7521	0.1051	0.113*	
F1	0.90279 (11)	1.1721 (4)	−0.07006 (17)	0.1125 (8)	
H1	0.6954 (14)	0.606 (4)	0.0454 (19)	0.059 (8)*	
N1	0.74101 (12)	0.5785 (4)	0.06106 (16)	0.0567 (6)	
O1	0.73060 (10)	−0.0227 (3)	0.26022 (14)	0.0667 (6)	
O2	0.83483 (9)	0.0669 (3)	0.23152 (13)	0.0584 (5)	
O3	0.42082 (9)	0.4306 (3)	0.10206 (13)	0.0618 (6)	

### Atomic displacement parameters (Å^2^)

**Table:**

	*U*^11^	*U*^22^	*U*^33^	*U*^12^	*U*^13^	*U*^23^
C1	0.0401 (13)	0.0536 (15)	0.0482 (14)	−0.0053 (12)	0.0170 (11)	0.0019 (13)
C2	0.0465 (15)	0.076 (2)	0.0642 (17)	−0.0017 (15)	0.0135 (13)	0.0221 (16)
C3	0.0434 (15)	0.093 (2)	0.082 (2)	−0.0056 (16)	0.0193 (15)	0.026 (2)
C4	0.0596 (18)	0.073 (2)	0.082 (2)	−0.0085 (16)	0.0371 (16)	0.0172 (18)
C5	0.074 (2)	0.065 (2)	0.085 (2)	0.0109 (16)	0.0370 (17)	0.0304 (18)
C6	0.0450 (15)	0.0681 (19)	0.078 (2)	0.0063 (14)	0.0193 (14)	0.0157 (17)
C7	0.0403 (13)	0.0433 (14)	0.0443 (14)	−0.0067 (11)	0.0125 (10)	0.0005 (12)
C8	0.0456 (14)	0.0464 (15)	0.0548 (15)	−0.0018 (12)	0.0150 (12)	−0.0075 (13)
C9	0.0412 (13)	0.0540 (17)	0.0528 (15)	−0.0131 (12)	0.0081 (11)	−0.0127 (13)
C10	0.0361 (12)	0.0550 (16)	0.0482 (14)	−0.0008 (12)	0.0118 (11)	0.0038 (13)
C11	0.0490 (15)	0.0417 (14)	0.0668 (17)	−0.0045 (12)	0.0202 (13)	−0.0085 (13)
C12	0.0449 (14)	0.0364 (14)	0.0694 (18)	−0.0074 (11)	0.0164 (13)	−0.0033 (13)
C13	0.0364 (12)	0.0493 (15)	0.0502 (15)	−0.0021 (12)	0.0097 (11)	0.0004 (13)
C14	0.0410 (13)	0.0406 (14)	0.0471 (14)	−0.0060 (11)	0.0133 (11)	−0.0046 (12)
C15	0.0441 (13)	0.0422 (14)	0.0521 (15)	−0.0065 (12)	0.0174 (12)	−0.0088 (13)
C16	0.0616 (17)	0.0523 (17)	0.076 (2)	0.0087 (15)	0.0087 (15)	0.0069 (16)
C17	0.0565 (18)	0.093 (3)	0.104 (3)	0.0198 (18)	0.0101 (18)	0.004 (2)
C18	0.0521 (16)	0.091 (2)	0.085 (2)	0.0079 (16)	0.0267 (16)	−0.0201 (19)
F1	0.0896 (13)	0.1078 (17)	0.154 (2)	−0.0112 (12)	0.0592 (14)	0.0556 (15)
N1	0.0359 (12)	0.0658 (15)	0.0665 (15)	0.0010 (12)	0.0146 (11)	0.0177 (13)
O1	0.0589 (11)	0.0540 (12)	0.0889 (15)	−0.0014 (10)	0.0272 (11)	0.0181 (11)
O2	0.0439 (10)	0.0583 (12)	0.0718 (13)	0.0047 (9)	0.0177 (9)	0.0084 (10)
O3	0.0399 (10)	0.0733 (13)	0.0714 (13)	0.0012 (9)	0.0177 (9)	−0.0061 (11)

### Geometric parameters (Å, °)

**Table:**

C1—C6	1.368 (4)	C11—C12	1.385 (3)
C1—C2	1.377 (3)	C11—H11	0.9300
C1—N1	1.408 (3)	C12—H12	0.9300
C2—C3	1.375 (4)	C13—C14	1.347 (3)
C2—H2	0.9300	C13—N1	1.359 (3)
C3—C4	1.355 (4)	C13—H13	0.9300
C3—H3	0.9300	C14—C15	1.463 (3)
C4—C5	1.362 (4)	C15—O1	1.208 (3)
C4—F1	1.363 (3)	C15—O2	1.346 (3)
C5—C6	1.375 (4)	C16—O2	1.444 (3)
C5—H5	0.9300	C16—C17	1.499 (4)
C6—H6	0.9300	C16—H16A	0.9700
C7—C12	1.384 (3)	C16—H16B	0.9700
C7—C8	1.392 (3)	C17—H17A	0.9600
C7—C14	1.486 (3)	C17—H17B	0.9600
C8—C9	1.379 (3)	C17—H17C	0.9600
C8—H8	0.9300	C18—O3	1.425 (3)
C9—C10	1.378 (4)	C18—H18A	0.9600
C9—H9	0.9300	C18—H18B	0.9600
C10—O3	1.372 (3)	C18—H18C	0.9600
C10—C11	1.381 (3)	N1—H1	0.83 (2)
			
C6—C1—C2	118.7 (2)	C7—C12—H12	118.9
C6—C1—N1	119.1 (2)	C11—C12—H12	118.9
C2—C1—N1	122.2 (2)	C14—C13—N1	125.6 (2)
C3—C2—C1	120.5 (3)	C14—C13—H13	117.2
C3—C2—H2	119.8	N1—C13—H13	117.2
C1—C2—H2	119.8	C13—C14—C15	119.6 (2)
C4—C3—C2	119.2 (2)	C13—C14—C7	122.8 (2)
C4—C3—H3	120.4	C15—C14—C7	117.6 (2)
C2—C3—H3	120.4	O1—C15—O2	121.7 (2)
C3—C4—C5	121.9 (3)	O1—C15—C14	124.2 (2)
C3—C4—F1	118.8 (3)	O2—C15—C14	114.2 (2)
C5—C4—F1	119.3 (3)	O2—C16—C17	107.4 (2)
C4—C5—C6	118.3 (3)	O2—C16—H16A	110.2
C4—C5—H5	120.8	C17—C16—H16A	110.2
C6—C5—H5	120.8	O2—C16—H16B	110.2
C1—C6—C5	121.4 (2)	C17—C16—H16B	110.2
C1—C6—H6	119.3	H16A—C16—H16B	108.5
C5—C6—H6	119.3	C16—C17—H17A	109.5
C12—C7—C8	117.2 (2)	C16—C17—H17B	109.5
C12—C7—C14	121.8 (2)	H17A—C17—H17B	109.5
C8—C7—C14	120.9 (2)	C16—C17—H17C	109.5
C9—C8—C7	121.1 (2)	H17A—C17—H17C	109.5
C9—C8—H8	119.4	H17B—C17—H17C	109.5
C7—C8—H8	119.4	O3—C18—H18A	109.5
C10—C9—C8	120.5 (2)	O3—C18—H18B	109.5
C10—C9—H9	119.7	H18A—C18—H18B	109.5
C8—C9—H9	119.7	O3—C18—H18C	109.5
O3—C10—C9	115.8 (2)	H18A—C18—H18C	109.5
O3—C10—C11	124.6 (2)	H18B—C18—H18C	109.5
C9—C10—C11	119.6 (2)	C13—N1—C1	126.5 (2)
C10—C11—C12	119.2 (2)	C13—N1—H1	117.1 (19)
C10—C11—H11	120.4	C1—N1—H1	116.3 (19)
C12—C11—H11	120.4	C15—O2—C16	115.8 (2)
C7—C12—C11	122.3 (2)	C10—O3—C18	118.0 (2)
			
C6—C1—C2—C3	0.9 (4)	C10—C11—C12—C7	0.5 (4)
N1—C1—C2—C3	−178.1 (3)	N1—C13—C14—C15	177.8 (2)
C1—C2—C3—C4	0.1 (5)	N1—C13—C14—C7	−2.3 (4)
C2—C3—C4—C5	−1.3 (5)	C12—C7—C14—C13	−62.1 (3)
C2—C3—C4—F1	179.9 (3)	C8—C7—C14—C13	119.3 (3)
C3—C4—C5—C6	1.4 (5)	C12—C7—C14—C15	117.8 (3)
F1—C4—C5—C6	−179.8 (3)	C8—C7—C14—C15	−60.8 (3)
C2—C1—C6—C5	−0.8 (4)	C13—C14—C15—O1	179.5 (2)
N1—C1—C6—C5	178.2 (3)	C7—C14—C15—O1	−0.4 (4)
C4—C5—C6—C1	−0.3 (5)	C13—C14—C15—O2	0.3 (3)
C12—C7—C8—C9	1.2 (4)	C7—C14—C15—O2	−179.6 (2)
C14—C7—C8—C9	179.8 (2)	C14—C13—N1—C1	172.4 (2)
C7—C8—C9—C10	−0.1 (4)	C6—C1—N1—C13	176.2 (3)
C8—C9—C10—O3	179.7 (2)	C2—C1—N1—C13	−4.8 (4)
C8—C9—C10—C11	−0.7 (4)	O1—C15—O2—C16	−2.4 (3)
O3—C10—C11—C12	−179.9 (2)	C14—C15—O2—C16	176.8 (2)
C9—C10—C11—C12	0.5 (4)	C17—C16—O2—C15	−169.0 (2)
C8—C7—C12—C11	−1.3 (4)	C9—C10—O3—C18	174.3 (2)
C14—C7—C12—C11	−180.0 (2)	C11—C10—O3—C18	−5.2 (4)

### Hydrogen-bond geometry (Å, °)

**Table:**

*D*—H···*A*	*D*—H	H···*A*	*D*···*A*	*D*—H···*A*
C12—H12···O1^i^	0.93	2.49	3.401 (3)	167
N1—H1···O3^ii^	0.83 (2)	2.56 (3)	3.229 (3)	138 (2)
C16—H16B···Cg1^iii^	0.97	2.99	3.788 (3)	141
C18—H18A···Cg2^iv^	0.96	2.80	3.626 (3)	145

Symmetry codes: (i) *x*, *y*+1, *z*; (ii) −*x*+1, −*y*+1, −*z*; (iii) *x*, −*y*+1/2, *z*+1/2; (iv) −*x*+1, −*y*, −*z*.
